# Comparison of Three Different Approaches in Pediatric Gartland Type 3 Supracondylar Humerus Fractures Treated With Cross-Pinning

**DOI:** 10.7759/cureus.8780

**Published:** 2020-06-23

**Authors:** Abuzer Uludağ, Hacı Bayram Tosun, Talip Teoman Aslan, Öznur Uludağ, Abdussamed Gunay

**Affiliations:** 1 Orthopaedics, Adiyaman University Faculty of Medicine, Adiyaman, TUR; 2 Orthopaedics, Istanbul Medipol University, Istanbul, TUR; 3 Orthopaedics and Traumatology, Darıca Farabi State Hospital, Kocaeli, TUR; 4 Anesthesiology and Reanimation, Adıyaman University Faculty of Medicine, Adıyaman, TUR

**Keywords:** surgical treatment, supracondylar humeral fracture, pediatric fractures, percutaneous pinning

## Abstract

Introduction

Although closed reduction and percutaneous pinning are the accepted treatment approaches in pediatric humerus supracondylar fractures, the treatment approach in fractures without closed reduction remains unclear. This study compared the results of three different cross-pinning treatment methods.

Materials and methods

A total of 62 patients (1-13 years old) who were operated for Gartland type 3 humerus supracondylar fractures between 2007 and 2016 were evaluated retrospectively. Of the patients evaluated, 24 patients had closed reduction, 25 patients had direct reduction from the medial, and 13 patients had direct reduction from the lateral and cross-pinning. The functional and cosmetic results of the patients were evaluated according to Flynn's criteria. In addition, the Baumann angle, lateral capitellohumeral angle (LCHA), and postoperative complications were compared among groups.

Results

Both functional and cosmetic results and the Bauman and LCHA angles were similar in all three groups. In patients with open reduction, the control duration was significantly longer than that in patients with closed reduction, and this difference was due to a recent increase in the surgeons' preference for closed surgery. Two patients underwent pin site infection and two patients developed nerve palsy. Only the first patient who developed ulnar nerve palsy recovered during follow-up. Secondary surgery was applied to the other patient who developed brachial artery occlusion with ulnar and median nerve paralysis, and they recovered during follow-up. Three patients who underwent open surgery from the medial, along with the two patients who had undergone open surgery, developed pinhole infection. These patients were subsequently recovered with antibiotherapy without further complications. A patient who underwent open lateral surgery developed compartment syndrome and fasciotomy was performed.

Conclusion

Closed reduction and percutaneous pinning are generally accepted approaches in the treatment of pediatric humerus supracondylar type 3 fractures. However, in cases where closed reduction cannot be achieved, pinning with the medial approach and taking the ulnar nerve and medial colon is a reliable method to avoid both ulnar nerve injury and cubitus varus.

## Introduction

In the pediatric age group, supracondylar fractures of the humerus are the most common elbow fractures. Although they are common between the ages of two and eight years, the peak is between the ages of four and six. About 98% of these fractures are observed in extension type after a fall on an open arm. Closed reduction and percutaneous pinning are the preferred treatment methods for the surgical treatment of Gartland type 3 supracondylar humerus fractures in which posterior and anterior cortex contact is discontinued. However, there is no consensus on which of the open reduction methods is more advantageous in the surgical treatment of such fractures where closed reduction is not an option [1-6]. In this study, we compared the results of three different reduction methods in the surgical treatment of Gartland type 3 supracondylar humerus fractures.

## Materials and methods

Study population

The study was started after clinical research ethics committee approval was obtained (Date: 20.03.2019, Number: 25). A total of 104 patients aged between one and 13 years who underwent surgery in our clinic for a Gartland type 3 supracondylar humerus fracture between 2008 and 2017 were retrospectively analyzed. Patients with flexion type fractures, open fractures, patients with a secondary fracture on the same side, patients under the age of one and above the age of 13, patients with systemic diseases, and patients who were discontinued from being followed were excluded from the study. Thus, a total of 62 out of 104 patients were included in the study. Twenty-four patients underwent closed reduction and percutaneous pinning, 25 patients underwent open reduction with medial intervention and percutaneous pinning, and the remaining 13 patients underwent open reduction and percutaneous pinning with lateral intervention.

Operative treatment

All patients were operated under general anesthesia and within the first 24 hours of the incidence. Seventy-five mg/kg cefazolin sodium prophylaxis was applied to all patients preoperatively. After the reduction of the fracture under C-arm fluoroscopy control, percutaneous fixation was performed using 1.6 - 2.0 mm Kirschner (k) wires.

**Figure 1:**
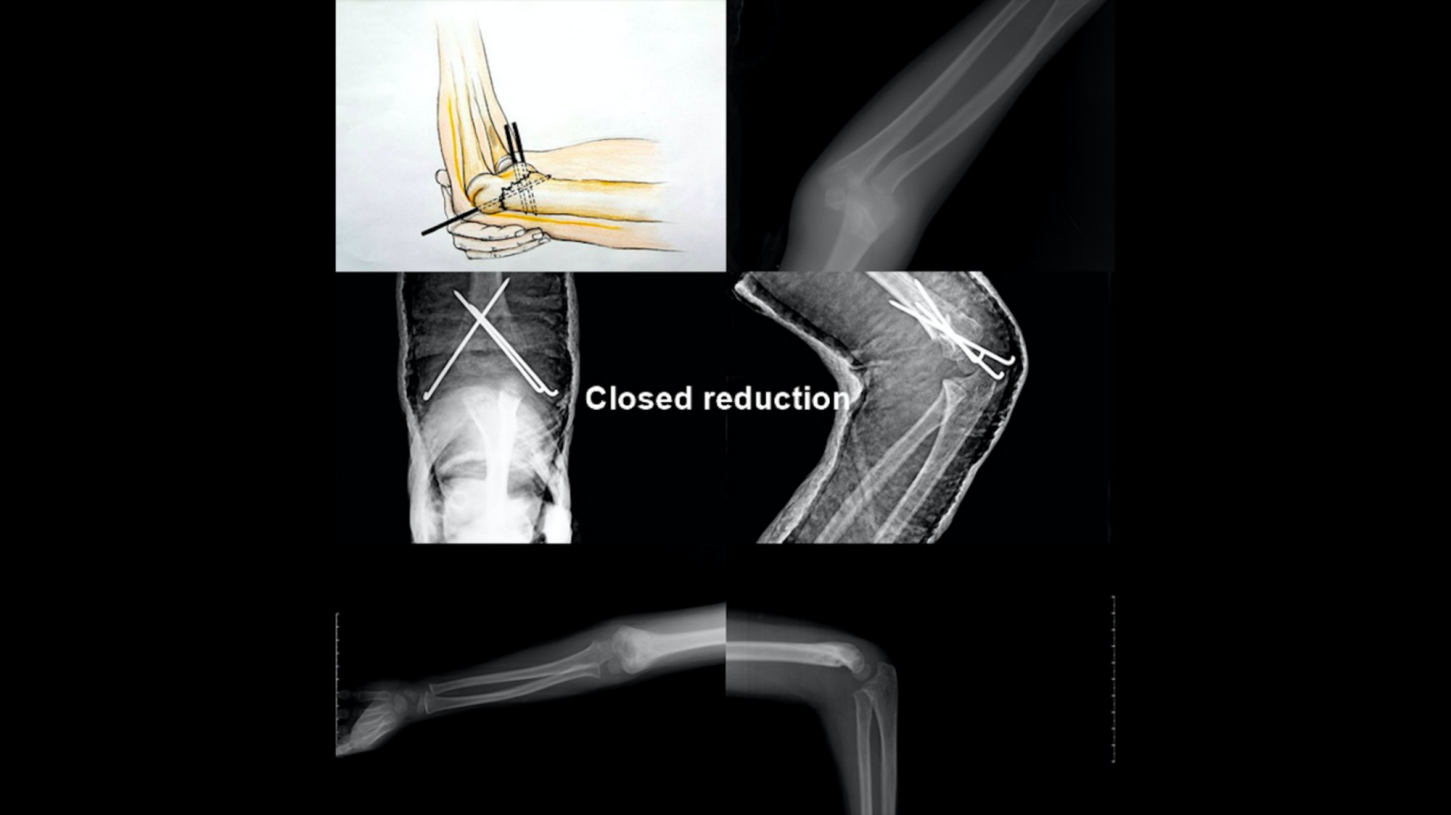
Closed Reduction

The patients with closed reduction who underwent lateral fixation with a k-wire in the fluoroscopy control were then subjected to a second k-wire with medial percutaneous pinning while elbow flexion was reduced by 600. Another k-wire was then applied laterally to increase stability (Figure 1).

**Figure 2:**
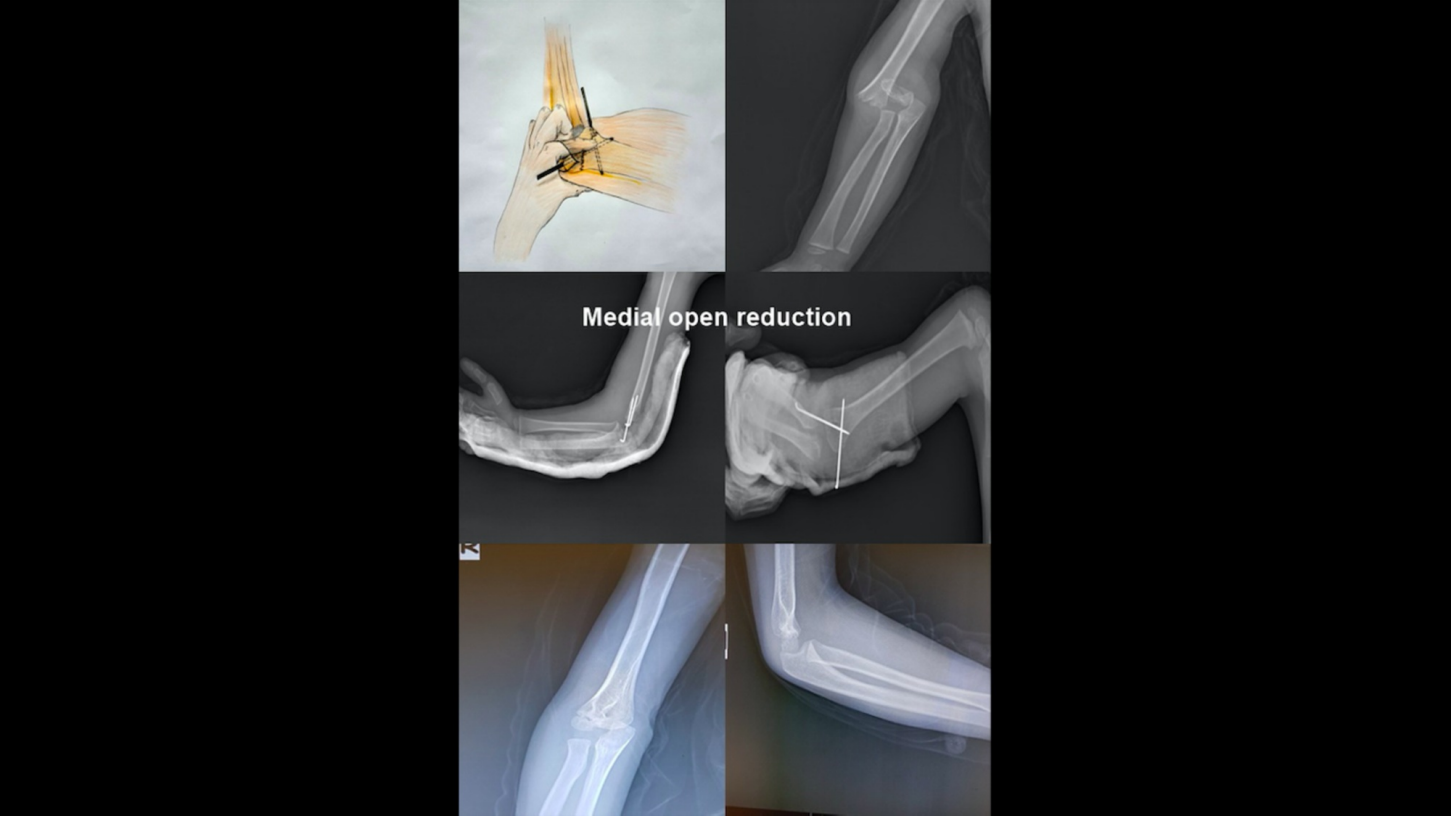
Medial Open Reduction

All patients who underwent open reduction using medial intervention had a 3 to 4 cm longitudinal incision over the elbow medial epicondyle by a single surgeon without a closed reduction maneuver trial. The skin and subcutaneous and deep fascia were passed and the ulnar nerve explored without removal from its canal. Then, the fracture line was reached while observing the medial colon under the brachialis and triceps muscles. The fracture is palpated with a finger and reduced. Under C-arm fluoroscopy control, percutaneous pinning was performed with a k-wire first from the medial and later from the lateral (Figure 2).

**Figure 3:**
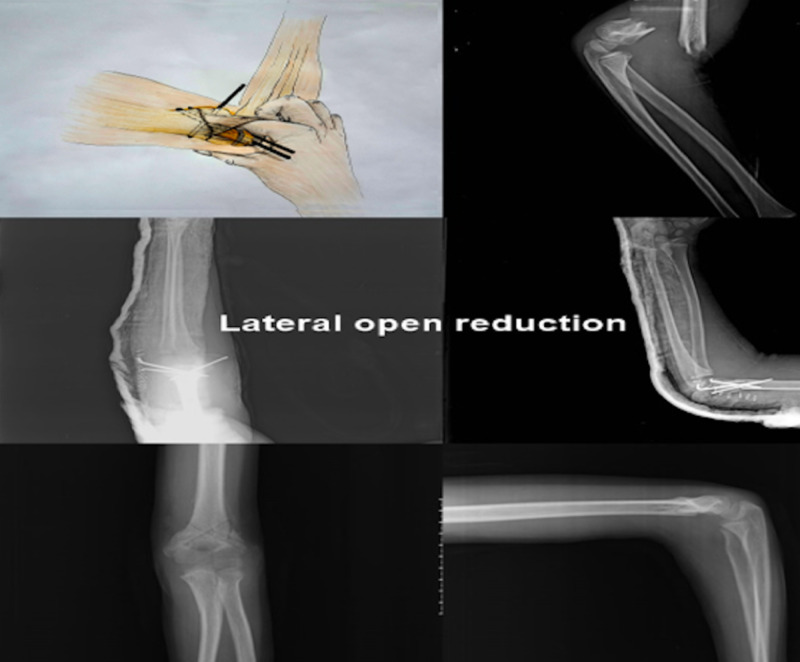
Lateral Open Reduction

Patients who underwent open reduction with lateral intervention had a 3-4 cm incision through the elbow lateral epicondyle without attempting a closed reduction maneuver by a single surgeon. After crossing the fascia, a lateral colon was seen between the triceps and brachioradialis muscles, and the fracture line was reached. Reduction was performed after the fracture was palpated. Three k-wire percutaneous pinnings were performed, Two of them were applied laterally and the other was applied medially when the elbow was flexed at 600 (Figure 3).

In all approaches, the reduction and fixation of the fracture were reconsidered under C-arm fluoroscopy, and the tips of the k-wires were bent over and trimmed to surpass the skin. The wound was duly closed in patients undergoing open reduction.

Postoperative follow-up

A long arm circular cast was applied to the patients who had closed reduction after surgery and a long arm plaster cast was applied to the patients who had an open reduction. On postoperative Day 1, patients were discharged after circulatory follow-up. Only patients in the open group were given 10 mg/kg cefuroxime axetil two times a day for three days after discharge. Controls were conducted on the fifth, 15th, and 30th postoperative days. In the fourth week, the cast was terminated and the k-wires were removed in the clinic. Active and passive elbow joint movements were started. The range of joint motion of the patients was checked at 10-day intervals. Patients with an inadequate range of motion at the postoperative second-month follow-up were referred to the physical therapy clinic.

The demographic characteristics along with the functional and cosmetic results gathered from the patients were evaluated according to Flynn criteria [7].

Evaluation of outcomes

The data were evaluated with SPSS (Statistical Package for the Social Sciences) 21.0 software (IBM, Armonk, NY). In the analysis of variables, the X2 and Kruskal-Wallis tests were used for non-parametric tests. Averages were given with standard deviations (mean ± SD). P <0.05 was accepted as statistically significant. 

## Results

A total of 62 patients out of 104 were included in the study. The mean age of the patients was 5.96 ± 2.78 (range, 1-13) years. While 52% of the patients undergoing closed reduction had female dominance, 65% of patients undergoing open reduction were males. The mean age in the closed reduction group was 5.79 ± 2.41 years, while the mean age in the open surgery group was 6.07 ± 3.02 years. The mean follow-up period was 4.25 ± 1.98 years in patients with closed reduction, while the mean follow-up period was 5.84 ± 2.53 years (p = 0.034). Complications were seen in 4 patients who underwent closed reduction (16.7%) and 7 patients (18.4%) underwent open reduction (Table 1).

**Table 1:** Comparison of three different surgical methods X: Two ulnar and one median nerve palsy; Y: Only ulnar nerve palsy

		Closed Reduction	Medial Approach	Lateral Approach	p-value
Patient	(n)	24	25	13	-
Age (Year)	(ort±SD)	5,79±2,41	6,32±3,17	5,61±2,78	0,673
Male/Female	(Ratio)	0.76	2.12	2.25	0,215
Baumann angle	(Degree	21,00±4,00	21,24±2,81	19,38±4,27	0,923
Lateral humeral condylar angle	(Degree)	38,95±8.70	39,36±5,67	39,53±7,38	0,923
Complications	(n)	4	2	5	0,068
Nerve injury	(n)	3^x^	0	1^y^	
Vascular injury	(n)	1	0	0	
Compartment syndrome	(n)	0	0	1	
Pin tract infection	(n)	2	2	3	

Two patients who underwent closed percutaneous pinning, three patients who underwent the medial approach, and two patients who underwent the lateral approach had pin infection. All patients recovered without any additional antibiotic treatment.

Ulnar nerve symptoms were observed in a patient undergoing closed reduction and with the removal of the k-wires at postoperative fourth week, the ulnar nerve symptoms completely disappeared after six months. In another patient who had undergone closed reduction, in the second postoperative month, there was a lack of filling in the radial nerve with ulnar and median nerve symptoms. Surgery was performed in the postoperative third month. It was observed that the median nerve was under compression by the bone fragment and the brachial artery was trapped between fracture fragments. Median nerve compression was removed and a release was applied to the ulnar nerve. The brachial artery was revascularized with a saphenous vein graft. In the postoperative fifth month, vascular and nerve functions were completely recovered.

In one patient who underwent open reduction with the lateral approach, pain and edema in the forearm and elbow and pain with passive extension of the fingers were observed at the third postoperative hour. The patient underwent fasciotomy because of the compartment syndrome and during the follow-up, the patient recovered without any problems.

According to Flynn's criteria, no significant difference was observed between all three groups in terms of functional and cosmetic properties (Tables 2-4). 

**Table 2:** Functional outcomes according to Flynn’s criteria

Satisfactory results	Closed Reduction	Medial Approach	Lateral Approach	p-value
Excellent (%)	100 (100)	23(92)	12(92.3)	0,375
Good (%)	0	2(8)	1(7.7)	
Fair (%)	0	0	0	
Poor (%)	0	0	0	

**Table 3:** Cosmetic outcomes according to Flynn’s criteria

Satisfactory Results	Closed Reduction	Open Reduction(Total)	Medial Approach	Lateral Approach	p-value
					Excellent n (%)	23(95.8)	31(81.6)	21(84)	10 (77)	0,103
Good n (%)	1(4.2)	6(15.8)	4(16)	2 (15.3)						
Fair n (%)	0	0	0	0						
Poor n (%)	0	1(2.6)	0	1(7.7)						

**Table 4:** Comparison of the open and closed reduction

		Total (Open and Closed)	Closed Reduction	Open Reduction (Medial and Lateral)	p-value
Patients	(n)	62	24	38	
Age (Year)	(ort±SD)	5,96±2,78(1-13)	5,79±2,41	6,07	0,805
Male/Female	ratio	1,33	0,76	1,92	0,080
Baumann angle (Degree)	(ort±SD)	20,75±3,64	21,00±4,00	20.60±3.44	0,441
Lateral humeral condylar angle (Degree)	(ort±SD)	39,24±7,21	38,95±8.70	39,42±6.21	0,720
Complications	(n)	11	4	7	0,861
Functional results	(n)	Excellent:59, Good:3	Excellent: 24	Excellent; 35, Good:3	0,161
Cosmetic results	(n)	Excellent:54 Good:7 Poor:1	Excellent:23 Good:1	Excellent: 31 Good:6 Poor:1	0,176

## Discussion

Supracondylar humerus fractures occur in the ration of 60-71/100000. Surgical intervention is required in about 16% of these fractures, most of which are conservatively treated [1]. About 17% of supracondylar humerus fractures consist of Gartland type 3 fractures [8]. In the treatment of these types of fractures, percutaneous fixation with cross-pinning is performed after closed reduction of the fracture [3-6]. The risk of ulnar nerve damage caused by the k-wire applied from medial when percutaneous cross-pinning is high, and the concern of not being able to provide biomechanical stability using only the lateral k-wire has caused controversy regarding pinning [8-11].

The percutaneous pinning configuration for the fixation of humerus supracondylar fractures can be performed as one medial and lateral cross-pin, two lateral pins only, two lateral and one medial pins, or three lateral pins. While biomechanical studies show the superiority of the cross-pin [12-13], clinical trials indicate no superiority [14]. Aslani et al. reported that stability was not achieved with two lateral pins in fractures distant from the olecranon fossa or in the medial arm, but stability was achieved in 27% of the patients after the insertion of an additional third pin from the lateral [15]. They stated that this type of fracture could provide complete stability with additional pinning from the medial. Reisoğlu et al. compared patients who underwent cross-pinning and lateral pinning [16]. Reduction loss was observed in 18.7% of the patients who underwent lateral pinning, while 7.6% of the patients who had cross-pinning had a loss of reduction. They stated that cross-pinning should be performed especially in patients with disintegration and instability in the medial colon. In a biomechanical study by Larson et al., they stated that medial fragmentation significantly reduced fracture stability and that the most stable pin configuration against torsion forces could be achieved by placing two lateral and one medial pin [17]. In our study, two lateral pins and one medial pin were used in the closed percutaneous pinning group and open lateral group.

In general, the surgeons' personal experience determines how and in which configuration k-wires are used. Carter et al. found that of the 309 pediatric orthopedic surgeons, 33% preferred two lateral pins, 33% preferred three lateral pins, and 30% preferred cross-pins [18]. Lee et al. reported that hand surgeons preferred a more stable pinning technique by using a triple pin, while pediatric orthopedic surgeons and general orthopedic surgeons were more inclined to use two pins on the lateral side [19]. We prefer cross-pinning, which is often shown to be more stable fixation in experimental studies. We think that other approaches in clinical applications have similar functional results with cross-pinning due to the fact that the plaster splint, which is used up to one month postoperatively, provides the restriction of rotational movements by reducing elbow movements. For this purpose, we think that it is beneficial to use cross-pinning, which is a more stable method in patients who do not have good cooperation or require constant dressing. Therefore, each supracondylar humerus fracture should be considered separately and the type of fixation should be determined according to the type of fracture, the location of disintegration, and the direction of displacement [12-14,20].

Open reduction is required since closed reduction cannot be achieved in 2%-12% of humeral supracondylar Gartland type 3 fractures. Medial, lateral, anterior, posterior, or double incision approaches can be used for open reduction. However, the use of these approaches is controversial [20-21].

The medial approach with open reduction has advantages such as preventing rotation via better observation of the medial column, preventing the formation of cubitus varus and ulnar nerve paralysis, and ulnar nerve expansions [11,22-24]. Eren et al. compared the patients who had cross-pinning after a lateral and medial intervention [25]. They reported that they achieved 100% good and excellent functional results in patients who underwent a medial intervention and 95% good and excellent functional results in patients who underwent lateral intervention. Barlas et al. performed a successful medial open reduction to patients with twice unsuccessful closed reduction attempts (23.25%) [26]. They emphasized the excellent exploration with the medial approach and the feasibility of the medial approach in non-reduced patients. In a review study by Mazzini et al., functional and cosmetic results were reported to be more successful than those with the anteromedial approach [21]. In our study, good and excellent cosmetic results were obtained in 100% of patients who underwent a medial intervention, while in 92.3% of patients undergoing lateral intervention indicated good and excellent results. We think that the medial approach is preferable because it allows for the reconstruction of the medial colon by providing better exploration.

Lateral open reduction is generally preferred because it is an easier approach to avoid ulnar nerve injury during exposure. However, changes in the angle of the elbow carriage may be a common complication of the lateral approach. Errors in the evaluation of reduction and medial tilt under fluoroscopy result in the formation of cubitus varus deformity. However, similar results in comparative studies in both approaches were reported in terms of functionally [21-25]. In our study, consistent with the literature, similar functional results were observed among the groups that underwent open reduction with both medial and lateral approaches.

Complications such as neurovascular injury, compartment syndrome, pin-tract infections, and cubitus varus may occur after a humeral supracondylar fracture. Ulnar nerve injury is a common complication of nerve injuries. The cross-pinning is indicated to cause four to five times more ulnar nerve injury than only lateral pinning. Most of them recover within a few months without requiring additional intervention [3-5,14-27]. Complication rates can be reduced with decreased flexion of the elbow or as seen of the ulnar nerve by applying a small incision during performing medial pinning [28]. In a meta-analysis performed with 1158 patients, Brauer et al. reported that 4.1% of the patients who underwent cross-pinning with k-wire were observed the ulnar nerve damage [27]. In our study, two out of 24 closed reduction patients who underwent cross-pinning had ulnar nerve damage. A single patient out of 13 cases who underwent the close medial pinning together with open lateral pinning was seen ulnar nerve damage. No ulnar nerve damage was observed in the medial open fixation method that observe the ulnar nerve. We conclude that the high number of ulnar nerve damage reported in the literature could be due to the lack of ability to observe the ulnar nerve during the medial pinning, excessive swelling of the elbow that prevents sufficient palpation of the medial epicondyle, and the insufficient reduction of elbow flexion. Ulnar nerve injuries may be caused by direct injury, nerve traps in the tunnel, or the soft tissues around the passed wire [14]. To avoid ulnar nerve injury in patients undergoing closed reduction, we recommend sending the medial pin after a mini-incision to observe the ulnar nerve, especially in patients with excessive swelling.

## Conclusions

In conclusion, the general treatment approach in Gartland type 3 humerus supracondylar fractures in the pediatric age group is closed reduction of the fracture and percutaneous cross-pinning. It is not clear with which approach open reduction should be performed in fractures that do not have closed reductions and require open reduction. In our study, although there is no difference between complications and functional improvement between the lateral open approach and the medial open approach, we recommend the medial open approach in fractures that do not have a closed reduction and require open reduction. This is because the medial approach is a good choice to avoid both cubitus varus and ulnar nerve injury by the restoration of the medial column in fractures without closed reduction. The facts that this study was performed retrospectively and the number of our cases was low are among the limitations of our study. We think that conducting prospective studies with a larger number of cases will contribute to clarifying the controversial issues of open reduction.
